# Accuracy of the VO_2peak_ prediction equation in firefighters

**DOI:** 10.1186/1745-6673-9-17

**Published:** 2014-04-28

**Authors:** Rachel E Klaren, Gavin P Horn, Bo Fernhall, Robert W Motl

**Affiliations:** 1 Department of Kinesiology & Community Health, University of Illinois at Urbana-Champaign, 233 Freer Hall, 906 S. Goodwin Avenue, Urbana, IL 61801, USA; 2 Illinois Fire Service Institute, University of Illinois at Urbana-Champaign, 11 Gerty Dr., Champaign, IL 61820, USA; 3 College of Applied Health Sciences, University of Illinois-Chicago, 808 South Wood Street, Chicago, IL, USA

**Keywords:** Validation, Firefighter, Fitness, Aerobic capacity, Evaluation

## Abstract

**Background:**

A leading contributing factor to firefighter injury and death is lack of fitness. Therefore, the Fire Service Joint Labor Management Wellness-Fitness Initiative (WFI) was established that includes a focus on providing fitness assessments to all fire service personnel. The current fitness assessment includes a submaximal exercise test protocol and associated prediction equation to predict individual VO_2peak_ as a measure of fitness. There is limited information on the accuracy, precision, and sources of error of this prediction equation. This study replicated previous research by validating the accuracy of the WFI VO_2peak_ prediction equation for a group of firefighters and further examining potential sources of error for an individual firefighters’ assessment.

**Methods:**

The sample consisted of 22 firefighters who completed a maximal exercise test protocol similar to the WFI submaximal protocol, but the test was terminated when firefighters reached a maximal level of exertion (i.e., measured VO_2peak_). We then calculated the predicted VO_2peak_ based on the WFI prediction equation along with individual firefighters’ body mass index (BMI) and 85% of maximum heart rate. The data were analyzed using paired samples *t*-tests in SPSS v. 21.0.

**Results:**

The difference between predicted and measured VO_2peak_ was -0.77 ± 8.35 mL•kg^-1^•min^-1^. However, there was a weak, statistically non-significant association between measured VO_2peak_ and predicted VO_2peak_ (R^2^ = 0.09, F(1,21) = 2.05, *p* = 0.17). The intraclass correlation coefficient (ICC = 0.215, *p* > 0.05) and Pearson (*r* = 0.31, *p* = 0.17) and Spearman (ρ = 0.28, *p* = 0.21) correlation coefficients were small. The standard error of the estimate (SEE) was 8.5 mL•kg^-1^•min^-1^. Further, both age and baseline fitness level were associated with increased inaccuracy of the prediction equation.

**Conclusions:**

We provide data on the inaccuracy and sources of error for the WFI VO_2peak_ prediction equation for predicting fitness level in individual firefighters, despite apparently accurate predictions for a group of firefighters. These results suggest that the WFI prediction equation may need to be reevaluated as a means of precisely determining fitness for individual firefighters, which may affect employment status, duty assignment, and overall life safety of the firefighter.

## 

Firefighting is an occupation that requires individuals to work in demanding and often times physically and psychologically stressful conditions [1-6]. Successful and safe job performance requires firefighters to maintain, among other critical factors, a high level of aerobic capacity (i.e., fitness). One of the leading contributing factors to firefighter injuries is lack of fitness [7]. Sudden cardiac death also accounts for close to half of all on-duty firefighter fatalities in the United States [8]. This cause of mortality has been linked, in part, to fitness level [9-12].

Accordingly, the International Association of Firefighters (IAFF) and the International Association of Fire Chiefs (IAFC) established the Fire Service Joint Labor Management Wellness-Fitness Initiative (WFI) in 1997 [13]. The WFI includes a focus on fitness assessments for all fire service personnel -- a firefighter is recommended to be at or above a minimal level of fitness indicative of the ability to successfully and safely perform firefighting duties.

The gold standard for measurement of cardiorespiratory fitness is a test of peak oxygen consumption (VO_2peak_) in the laboratory through open circuit spirometry [14]. However, this test requires expensive equipment, extensive professional expertise, and may require physician supervision [15]. An alternative method involves predicting VO_2peak_ using a submaximal exercise test and validated equation. Both the revised 2008 edition of the WFI and 2013 National Fire Protection Association (NFPA) 1582 standard medical program include a submaximal exercise test protocol to predict a firefighter’s VO_2peak_[16,17]. This submaximal exercise test is based on the Gerkin treadmill protocol which involves a warm-up of three minutes at 3 miles-per-hour (mph) followed by increases in ramp incline by 2% or speed by 0.5-mph every minute (i.e., Stage 1: 4.5-mph and 0% incline; Stage 2: 4.5-mph and 2% incline; Stage 3: 5.0-mph and 2% incline; Stage 4: 5.0-mph and 4% incline; Stage 5: 5.5-mph and 4% incline; Stage 6: 5.5-mph and 6% incline; etc). The test is terminated when the participant reaches 85% of estimated maximum heart rate, based on the Tanaka formula ((208 – (0.7 × age)) × 0.85) [18]. The predicted VO_2peak_ value is then calculated from the test time (TT) required to achieve 85% of maximum heart rate and Body Mass Index (BMI) of the participant.

Previous research has assessed the accuracy of the 2008-revised WFI assessment for predicting VO_2peak_[19]. That study compared data from 63 male firefighters who performed both submaximal and maximal WFI exercise tests with expired gases analyzed by a CardioCoach CO_2_™ portable metabolic system during the maximal test. Data analysis (i.e., *t*-test) demonstrated no statistically significant difference between the predicted and measured VO_2peak_ values, suggesting that VO_2peak_ values from the submaximal protocol accurately reflect directly measured VO_2peak_. This result was deemed to be an improvement over the previous version of the WFI protocol that utilized different means of determining maximum heart rate (220-age) and the ACSM metabolic equation for running to predict VO_2peak_. The previously accepted approach has consistently over predicted aerobic capacity and is no longer recommended in predicting VO_2peak_ in individual firefighters [20].

The purpose of our study was to replicate previous research by cross-validating the WFI VO_2peak_ prediction equation. We further aimed to identify potential sources of error that may influence the accuracy of the prediction. We are unaware of research examining sources of error in estimation (i.e., participant age or fitness level) using the 2008-revised WFI equation. Lastly, we assessed the classification accuracy of the VO_2peak_ prediction equation using the WFI criterion of 42 mL•kg^-1^•min^-1^ as the absolute minimal level of fitness for duty (i.e., VO_2peak_) recommended for all firefighters, regardless of age and sex. This replication and confirmation of validity and accuracy of the equation is important as fire departments across the nation use the WFI protocol to predict VO_2peak_ and further require a minimal level of aerobic fitness in all firefighters as a requirement for employment or return to duty assignment. A lack of precision in the VO_2peak_ prediction equation could erroneously deny an individual firefighter from duty or place a firefighter on duty whose limited aerobic capacity may prevent them from appropriately carrying out demanding occupational duties and even present a risk for on-duty injury or cardiac death.

## Materials and methods

### Participants

Participants were limited to currently employed and active line firefighters and Illinois Fire Service Institute (IFSI) field staff who were a) between the ages of 18 and 60 years, b) cleared by their home department to participate in live-fire activities, c) free from known cardiovascular disease (as determined by the Participant Activity Readiness Questionnaire (PAR-Q [21], d) with no history of neurological, gait or postural disorder, and e) not recently suffering an injury or surgery that results in gait or postural disruption. All firefighters provided informed consent and associated procedures were approved by the University of Illinois institutional review board.

### Equipment

#### COSMED K4b2

The COSMED K4b2 is a commercially available portable metabolic unit that measures oxygen consumption (VO_2_) and carbon dioxide production (VCO_2_) on a breath-by-breath basis (K4b2 Cosmed, Italy). The K4b2 portable unit and battery weigh about 1100 grams (~2.4 pounds) and is specifically designed to be worn by the subject during activity [22]. The K4b2 uses an O_2_ and CO_2_ analyzer connected to a flowmeter with a bidirectional digital turbine. The flowmeter is attached to a rubber facemask (Hans-Rudolph, Kansas City, MO) that is placed to tightly cover the participant’s mouth and nose. Although the K4b2 system is validated for VO_2_ measurements over a wide range of exercise intensities [22], previous studies have demonstrated a repeatable pattern of overestimation [23,24] that can be corrected by using a validated regression equation [24]. Therefore, we applied the equation proposed by Duffield, et al. to the VO_2peak_ values measured by the K4b2 [24]. After a 30-minute warm-up, the O_2_ and CO_2_ analyzers of the K4b2 were calibrated using previously verified concentrations of gases, and the flow meter was calibrated using a 3 L syringe (Hans Rudolph, Kansas City, MO). The K4b2 and battery were both placed in the standard shoulder harness that was secured with the K4b2 resting on the chest and the battery on the upper back. This standard harness allows for minimal interference during ambulation on the treadmill.

### Maximal exercise test procedure

All maximal exercise tests were performed at the Illinois Fire Service Institute (IFSI) in Urbana-Champaign, IL. A research member initially measured the firefighter’s height and weight using a standard weight scale and height rod. Each test began with 5-minute period of data collection in the sitting position to allow for the collection of resting heart rate and oxygen consumption data. Participants then began walking on the treadmill for a 3-minute warm-up period. After the warm-up, firefighters completed the same Gerkin treadmill protocol used in the WFI submaximal assessment. However, the test was not terminated when firefighters reached 85% of estimated maximum heart rate, but rather was terminated when firefighters reached a maximal level of exertion. Verbal encouragement was provided throughout the testing session by research staff to ensure maximal effort. At each minute, heart rate and rating of perceived exertion (RPE) [25] were recorded. The Borg RPE scale was described to each participant prior to testing to allow for complete understanding and familiarization. The test was considered finished when the participant indicated volitional fatigue, and this coincided with a reported RPE ≥17. There were no other criteria for completion such as plateau of VO_2_. A cool down period then followed, consisting of walking at a comfortable speed and 0% grade. The highest 15-second average recording of VO_2_ by the COSMED K4b2 was considered VO_2peak_.

### Data analysis

We initially calculated the predicted VO_2peak_ for each firefighter based on the WFI estimation equation [16]:

$$\begin{array}{l} \mathrm{Predicted}\;VO_{2\mathrm{peak}}\left(\mathrm{mL}•kg^{-1}•\mathrm{mi}n^{-1}\right)\\ \;=56.981+\left(1.242\times \mathrm{TT}\right)–\left(0.805\times \mathrm{BMI}\right). \end{array}$$

The test time (TT) wherein a firefighter reached 85% of estimated maximum heart rate (i.e., (208 – (0.7 × age)) × 0.85) was based on the K4b2 15-second averaging data as the time when the participant reached the intended heart rate value for 15 seconds and did not further decrease during the remainder of the test. This test time was then inserted into the WFI equation, along with BMI, to calculate predicted VO_2peak_. We then calculated the corrected measured VO_2peak_ by applying Duffield et al.’s regression equation to the recorded maximalVO_2peak_ from the K4b2. This equation is as follows [23]:

$$VO_{2\mathrm{peak},\mathrm{Measured}=}0.926\;\left(VO_{2\mathrm{peak},K4b2}–0.227\right).$$

All analyses were conducted using SPSS v. 21.0. Descriptive statistics are presented as mean ± SD. Paired samples *t-*tests with 2-tailed α of .05 were conducted for examining absolute mean differences in predicted vs. measured VO_2peak_. We estimated the association between the predicted and measured VO_2peak_ by using the Pearson product-moment correlation coefficient (*r*) and Spearman’s *ρ*. The scatterplot along with line of best fit and 95% confidence intervals is provided in a figure to visually demonstrate the association between predicted and measured VO_2peak_. We estimated the intraclass correlation coefficient (ICC) between predicted and measured VO_2peak_. Linear regression analysis was conducted by regressing predicted VO_2peak_ on measured VO_2peak_ in the entire sample to provide the R^2^ value for strength of association and standard error of the estimate (SEE) as an indication of precision. We produced a Bland-Altman plot of the difference between measured and predicted VO_2peak_ and mean of measured and predicted VO_2peak_. We examined the correlation of participant characteristics (for example, age, BMI, and measured fitness level) with the difference between predicted and measured VO_2peak_. The classification accuracy of the VO_2peak_ prediction equation was determined by identifying if the predicted VO_2peak_ value demonstrated an underestimation, overestimation, correct pass, or correct fail when compared to the measured VO_2peak_. Firefighters were classified according to the current aerobic fitness classification criterion used by the WFI and NFPA 1582 (42 mL•kg^-1^•min^-1^).

## Results

### Sample characteristics

The participants (n = 22) had an age range between 19 – 43 years with a mean of 27.5 years ± 7.1. The mean ± SD height, weight, and BMI of the participants were 1.82 ± 0.07 m, 89.6 ± 13.8 kg, and 27.1 ± 3.6 kg•m^2-1^, respectively.

### Descriptive statistics for measured and predicted VO_2peak_

The predicted VO_2peak_ (43.72 ± 3.60 mL•kg^-1^•min^-1^) was not significantly (*t* = -.430, *p* = 0.672) different than the measured VO_2peak_ (44.49 ± 8.72 mL•kg^-1^•min^-1^). The difference between predicted and measured VO_2peak_ was -0.77 ± 8.35 mL•kg^-1^•min^-1^. The intraclass correlation coefficient (ICC) between measured and predicted VO_2peak_ was weak (ICC = 0.215, *p* > 0.05). Pearson (*r* = 0.31, *p* = 0.17) and Spearman (*ρ* = 0.28, *p* = 0.21) correlation coefficients demonstrated weak associations between predicted and measured VO_2peak_.

### Regression analysis

**Figure 1:**
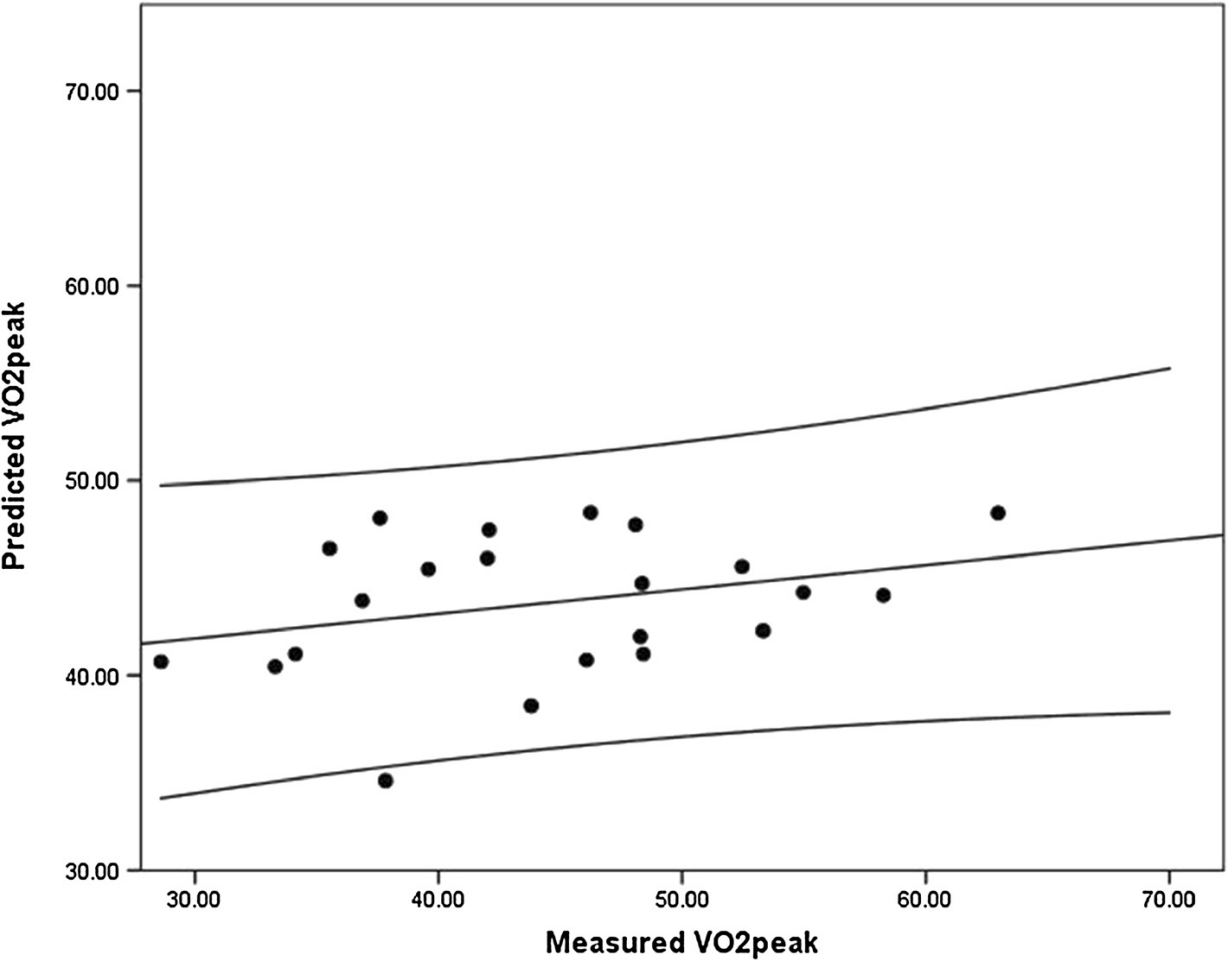
**Scatterplot along with line of best fit and 95% confidence limits for the association between measured and predicted VO**_**2peak**_**.**

The scatterplot along with the 95% confidence limits of the association between measured VO_2peak_ (independent variable) and predicted VO_2peak_ (dependent variable) for the overall sample is provided in Figure 1. There was a weak, statistically non-significant association between measured VO_2peak_ and predicted VO_2peak_ (R^2^ = 0.09, F(1,21) = 2.05, *p* = 0.17). The lack of precision for predicted vs. measured VO_2peak_ is demonstrated in the standard error of the estimate (SEE = 8.5 mL•kg^-1^•min^-1^).

### Bland-Altman plot

**Figure 2:**
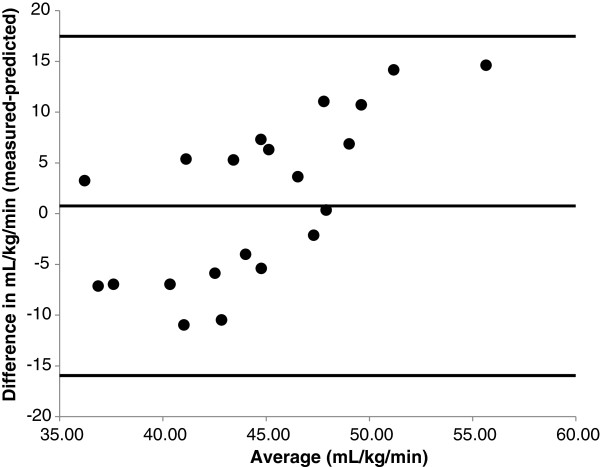
**Bland-Altman plot of the difference between measured and predicted VO**_**2peak **_**as a function of the mean of measured and predicted VO**_**2peak **_**in the entire sample.** The lines represent average ±2SDS.

The Bland-Altman plot in Figure 2 demonstrated variability in the prediction of VO_2peak_ in the overall sample, but this variability was within ±2SDs of the mean value. However, the difference between predicted and measured VO_2peak_ appeared to be directly related with the average VO_2peak_ value.

### Correlates of inaccuracy

There was a weak association between age and difference of predicted and measured VO_2peak_ values (*r* = -.36, *p* = 0.10; ρ = -.46, *p* < 0.05). There was a strong and significant association between fitness level (i.e., measured VO_2peak_) and difference between predicted and measured VO_2peak_ values (*r* = 0.91, *p* ≤ 0.05; ρ = 0.94, *p* ≤ 0.05).

### Classification accuracy

The VO_2peak_ prediction equation misclassified eight of the 22 firefighters (i.e., 36% of the sample) in comparison to measured VO_2peak_ values. Four participants’ VO_2peaks_ were underestimated (-6.07 ± 0.95 mL•kg^-1^•min^-1^), and four participants’ VO_2peaks_ were overestimated (8.58 ± 2.54 mL•kg^-1^•min^-1^), when using values calculated by the prediction equation. This is demonstrated below in Table 1.

**Table 1:** **Classification accuracy of the VO**_**2peak **_**prediction equation**

		**Measured VO**_**2peak**_	
		**‘Not Fit’**	**‘Fit for Duty’**
		**< 42 ml.kg**^**-1**^**.min**^**-1**^	**≥42 ml.kg**^**-1**^**.min**^**-1**^
**Predicted VO**_**2peak**_	**‘Not Fit’**	**N = 4**	**N = 4**
	**<42 ml.kg**^**-1**^**.min**^**-1**^	Measured: 33.46 ± 3.79	Measured: 46.64 ± 2.17
		Predicted: 39.20 ± 3.09	Predicted: 40.57 ± 1.51
	**‘Fit for Duty’**	**N = 4**	**N = 10**
	**≥42 ml.kg**^**-1**^**.min**^**-1**^	Measured: 37.39 ± 1.69	Measured: 50.87 ± 6.81
		Predicted: 45.97 ± 1.79	Predicted: 45.89 ± 2.07

## Discussion

We found no mean difference between predicted and measured VO_2peak_ at the overall group level, consistent with previous research [19]. We demonstrate a mean VO_2peak_ difference of -0.77 mL•kg^-1^•min^-1^ and previous research reported a mean difference of 0.25 mL•kg^-1^•min^-1^[19]. This might suggest that the 2008-revised WFI prediction equation is accurate. However, there was large error and disagreement in prediction at the individual level based on the SD of the mean difference between predicted and measured VO_2peak_, standard error of the mean (SEM), and the Bland-Altman plot. Further, there was a weak association between predicted and measured VO_2peak_ values based on correlation, ICC, and regression.

Although the difference between predicted and measured VO_2peak_ was small, the associated SD was ~ ± 9 mL•kg^-1^•min^-1^. This SD demonstrates high variability in accuracy when using the VO_2peak_ prediction equation. The Bland-Altman plot demonstrates evidence of systematic error between measured and predicted VO_2peak_ as a function of the mean of measured and predicted VO_2peak._ Some of the data points approach ~ 2 SDs difference, revealing high variability in the accuracy of the prediction equation based on firefighter’s baseline fitness level. Further, both Pearson and Spearman correlation coefficients demonstrated weak associations between predicted and measured VO_2peak_. The small ICC between measured and predicted VO_2peak_ (ICC = 0.215) highlights disagreement between measured and predicted values in such that participants’ rank of VO_2peak_ greatly differed depending on the measured or predicted value. Lastly, the statistically non-significant association between measured VO_2peak_ and predicted VO_2peak_ is demonstrated in the linear regression, and the lack of precision for predicted vs. measured VO_2peak_ is verified in the large standard error of the estimate (SEE = 8.5 mL•kg^-1^•min^-1^). This lack of relationship does not appear to be related to a truncated range of VO_2peak_ values, as our data indicate a measured VO_2peak_ range of 34 mL•kg^-1^•min^-1^.

The data analysis identified specific correlates of inaccuracy for the difference between predicted and measured VO_2peak_ values. We demonstrated age of the firefighters to be related to inaccuracy of the prediction such that the VO_2peak_ of older firefighters was recurrently overestimated and the VO_2peak_ of younger firefighters was underestimated when compared to measured VO_2peak_ values. This highlights a problem with the prediction equation for VO_2peak_ as fire departments in the United States employ men and women of wide age range, with the majority being younger than 50 years of age [26]. The prediction equation also consistently overestimated fitness in firefighters with a lower baseline fitness level (i.e., VO_2peak_) and underestimated firefighters’ fitness in those with a higher baseline fitness level. This association is further demonstrated in the Bland-Altman plot. Therefore, these inaccuracies may restrict younger firefighters with sufficient VO_2peak_ from being placed on duty. Importantly, together these correlates suggest the highest risk for overestimating fitness lies in older, less fit firefighters; the group that is at the highest risk for sudden cardiac events. Further, when classifying firefighters as fit for duty according to the WFI criterion (VO_2peak_ ≥ 42 mL•kg^-1^•min^-1^), the predicted VO_2peak_ values calculated from the estimation equation would misclassify eight firefighters, overestimating four and underestimating four firefighter’s actual VO_2peak_ (i.e., 36% of our sample would be misclassified). Therefore, four firefighters would be placed on duty with limited aerobic capacity, potentially increasing risk for inability to complete duty assignment or more importantly, on-duty injury or death. On the contrary, four firefighters with a suitable VO_2peak_ (i.e., VO_2peak_ ≥ 42 mL•kg^-1^•min^-1^) may be restricted from duty due to inaccurate VO_2peak_ predictions.

We did not have large enough sample for generating a new estimation equation, and this could be the focus of future research. One such revision might be to utilize a measure other than BMI in the estimation equation, since younger, resistance-trained participants with more lean muscle mass may have a higher BMI value, although the individual firefighter is not obese. Therefore, the use of body composition or girth might provide a more accurate estimation.

### Strengths and limitations

The strengths of our study include the wide age range of participants (range = 19 – 43 years) and the same research team and equipment conducted all tests for increased test consistency and inter-rater reliability. Further, each maximal exercise test was conducted by trained personnel with years of experience conducting maximal exercise tests with the COSMED K4b2 portable metabolic unit. Although this study has many strengths, it is not without limitations. A study limitation includes the small, predominantly male (n = 21) and Caucasian (n = 22) sample size. However, the US Department of Labor reported in 2002 that female firefighters accounted for 3.7% of all persons in the occupation [27], which provides rationale for the mostly male sample. Nevertheless, the findings of this study should be replicated in a different, larger sample of firefighters.

## Conclusion

This study demonstrates no overall or mean level inaccuracy for the 2008-revised WFI VO_2peak_ prediction equation compared with measured VO_2peak_ for the entire sample. However, we did demonstrate inaccuracy and variability in the estimation equation as a function of individual characteristics, particularly baseline fitness level and age of the firefighters. We further indicate that based on the WFI criterion minimum VO_2peak_ of 42 mL•kg^-1^•min^-1^, 36% of our sample of firefighters would be misclassified in terms of “fitness for duty”. These results suggest that the currently utilized prediction equation may need to be reevaluated as a means of precisely determining fitness for individual firefighters, which may affect employment status, duty assignment, and overall life safety of the firefighter. The need to accurately assess fitness for duty in the Fire Service is well documented and well founded, so continued development of a validated, accurate and precise fitness test is strongly encouraged.

## Abbreviations

WFI: Wellness-fitness initiative; BMI: Body mass index; IAFF: International Association of Firefighters; IAFC: International Association of Fire Chiefs; VO2peak: Peak of oxygen consumption; NFPA: National Fire Protection Association; mph: miles-per-hour; TT: Test time; IFSI: Illinois Fire Service Institute; PAR-Q: Participant Activity Readiness Questionnaire; RPE: Rating of perceived exertion; ICC: Intraclass correlation coefficient; SEE: Standard error of the estimate; SEM: Standard error of the mean.

## Competing interests

The authors declare that they have no competing interests.

## Authors’ contributions

GPH and RWM contributed to the conception and design of the study and interpretation of data. REK carried out all acquisition of data through maximal exercise testing sessions. BF was involved in data analysis and interpretation of data. All authors read and approved the final manuscript.
